# Virtually Naked: Virtual Environment Reveals Sex-Dependent Nature of Skin Disclosure

**DOI:** 10.1371/journal.pone.0051921

**Published:** 2012-12-26

**Authors:** Anna M. Lomanowska, Matthieu J. Guitton

**Affiliations:** 1 Institut Universitaire en Santé Mentale de Québec, Quebec City, Quebec, Canada; 2 Faculty of Medicine, Laval University, Quebec City, Quebec, Canada; ICREA-University of Barcelona, Spain

## Abstract

The human tendency to reveal or cover naked skin reflects a competition between the individual propensity for social interactions related to sexual appeal and interpersonal touch versus climatic, environmental, physical, and cultural constraints. However, due to the ubiquitous nature of these constraints, isolating on a large scale the spontaneous human tendency to reveal naked skin has remained impossible. Using the online 3-dimensional virtual world of Second Life, we examined spontaneous human skin-covering behavior unhindered by real-world climatic, environmental, and physical variables. Analysis of hundreds of avatars revealed that virtual females disclose substantially more naked skin than virtual males. This phenomenon was not related to avatar hypersexualization as evaluated by measurement of sexually dimorphic body proportions. Furthermore, analysis of skin-covering behavior of a population of culturally homogeneous avatars indicated that the propensity of female avatars to reveal naked skin persisted despite explicit cultural norms promoting less revealing attire. These findings have implications for further understanding how sex-specific aspects of skin disclosure influence human social interactions in both virtual and real settings.

## Introduction

Exposed naked skin serves as a biologically-relevant signal for human social interactions [1]–[4]. For instance, the appearance of skin is an inherent factor in human mate selection and disclosing naked skin plays an important role in sexual attractiveness [5]–[7]. Skin also acts as a medium for nonverbal communication through interpersonal touch [8], [9]. Conversely, covering naked skin is needed for protection from harmful climatic conditions, such as excessive cold, precipitation, or sun exposure, and damaging environmental stimuli, such as contact with rough surfaces and sharp objects, or insect bites. Furthermore, individual physical traits, such as body shape and skin appearance can influence one’s decision to cover or expose naked skin. Similarly, cultural norms contribute to variation in skin covering behavior across different populations [10]. The opposition between the human tendency to reveal or cover naked skin thus reflects a competition between the propensity for interpersonal interactions versus various external climatic, environmental, physical, and cultural constraints. Due to the ubiquitous nature of these external variables, it has remained impossible to isolate on a large scale the spontaneous human tendency to reveal naked skin.

The recent emergence of user-developed 3-dimensional (3D) virtual spaces accessible via the Internet provides a unique opportunity to address this problem. These massively multi-user settings, where individuals can interact using human-like 3D graphical representations of themselves (“avatars”), offer a tool to examine human behavior unhindered by real-world climatic conditions, environmental stimuli, or one’s existing physical form and appearance [11], [12]. In particular, the highly popular virtual world of Second Life (www.secondlife.com) is useful in this line of inquiry as it affords individuals the complete freedom to design the appearance and attire of their avatars without conforming to a predefined narrative [11], [13], [14]. Having an entirely original avatar is one of the main features of Second Life and users can fully customize the avatar’s body shape, skin, and clothes by selecting from the countless items created by other users and available either for free or for sale [15]. Users also have the option of creating their own clothes or modifying clothes made by others. Hence, in contrast to other multi-user 3D virtual worlds such as World of Warcraft, where clothing items are designed and generated by the system operators, all of the clothing in Second Life is created by the users inhabiting this virtual world. This feature of Second Life makes this virtual world particularly useful for examining spontaneous, user-generated skin covering and skin disclosure tendencies.

Here we used the virtual setting of Second Life as a testing ground to examine the degree to which humans spontaneously reveal naked skin when unconstrained by conventional climatic, environmental, and physical limitations. As naked skin plays a role in human sexual attractiveness [5]–[7], we further compared differences in skin exposure between virtual males and virtual females in this context. Finally, although the community of Second Life on the whole does not adhere to any explicit cultural norms, sub-communities following particular cultural practices exist within the context of role-play. We took advantage of a popular role-play community reenacting the fictional universe of Star Wars to specifically examine the contribution of cultural influences to skin exposure among virtual males and females. Our results indicate that virtual females reveal considerably more skin than virtual males.

## Methods

### Avatar Collection

Whole body images of 404 user-controlled avatars (192 males and 212 females) were collected in randomly selected public spaces of Second Life between January 2011 and January 2012. The virtual world of Second Life is divided into a grid of thousands of square regions of virtual land [13]. Public spaces were defined as all regions of the virtual world accessible to all adult users (18 years or older) that contained at least two avatars at the time of selection. Regions specifically identified as role-play areas were excluded. Different points on the Second Life world map were selected randomly and regions corresponding to these points were then visited using an observer avatar to determine their eligibility. If a region met the above criteria, images of avatars surrounding the observer avatar were captured. Avatars were only considered if they portrayed adult male or female humans. As more experienced users are likely to have more control over the amount of skin they wish to display due to greater familiarity with controlling the virtual interface and a larger choice of items in their virtual inventory, only avatars who were at least 90 days old were selected (avatar age is displayed in a public profile).

### Skin Disclosure Analysis

The percentage of naked areas of skin in relation to clothed areas was quantified according to the Lund and Browder chart, which divides the total surface of the skin into proportional areas representing body parts. The Lund and Browder chart was developed to efficiently assess the amount of skin damage in burn victims within the context of emergency hospital care [16]. The chart provides a tool that can be easily used by different evaluators with a high degree of reliability and consistency [17] and it is still commonly used in clinical settings [17], [18].

The Lund and Browder chart divides the front and back of the body into a total of 33 areas and assigns a percentage of skin represented by each area. In order to optimize the objective evaluation of skin coverage, we subdivided some of the larger areas represented on the chart (e.g., upper and lower legs, torso) to obtain a total of 60 areas. A single coder examined images of the front and back of the body of each avatar. As blind coding with regards to avatar sex was inherently not possible, several measures were undertaken to assure the objectivity of the assessment. First, in order to avoid the possibility of bias towards a particular outcome, there was no directional hypothesis regarding sex differences and all coding was completed before statistical analyses were conducted. Second, the coder was limited to selecting one of five options for each of the 60 areas of the body; fully covered, three-quarters covered, half covered, one-quarter covered or completely uncovered. Third, the individual body areas of all the avatars were coded first, before calculating the percentage of exposed naked skin. For each avatar, the score for each body area was converted into a representative percentage of the body based on the Lund and Browder chart. The total percentage of exposed skin of the entire body was then obtained by adding the percentage scores of all 60 areas of the body. Areas of the body covered by sheer or transparent clothing were also noted. A second measure of the percentage of exposed skin was obtained by excluding the head and hands, areas of skin typically exposed for communication and sensory functions. To assess intra-rater reliability, a random sample of 10% of the collected avatars was selected and their skin coverage was coded a second time. The mean difference between original and recoded scores was 1.17% with the 95% confidence interval between 0.77–1.58%.

### Contribution of Users’ Sex

The design of this study was strictly observational in order to obtain a purely random sample of avatars and avoid any selection or self-selection bias associated with recruitment of participants. Although no demographic information about the users behind the avatars was obtained, we used a statistical approach in order to estimate how the sex of the users contributed to the differences in exposed naked skin between male and female avatars. Previous work demonstrated that less than 25% of users of online virtual worlds, including Second Life, take on the opposite sex for their avatars [19]. After finding a significant difference in skin exposure between the entire sample of male and female avatars, we conducted a subsequent analysis that excluded the top 25% of female avatars that exposed most skin (excluded n = 53, leaving n = 159) and the bottom 25% of male avatars that exposed least skin (removed n = 48, leaving n = 144). This approach provides a conservative assessment of whether sex swapping on its own was responsible for the differences we observed between male and female avatars.

### Contribution of Avatar Body Shape

Human bodies are often portrayed in a hypersexualized manner in virtual spaces and related media, particularly with exaggerated and unrealistic body shape proportions [20]. To address the possibility that hypersexualized virtual portrayal of body shapes may contribute to the tendency to disclose naked skin in the setting we studied, we considered avatar body proportions relevant to male and female sexual attractiveness [21]–[24]. For instance, smaller waist to chest ratios in females and larger shoulder to hip ratios in males are associated with greater sexual attractiveness. We calculated waist to hip, waist to chest, waist to shoulder, and shoulder to hip ratios for both the front and back of the bodies of both male and female avatars using the collected images. Measurements of body shape were only obtained when the areas of interest were not obstructed by loose clothing and when the positioning of the torso and limbs did not interfere with accurate measurement of relevant body parts.

### Contribution of Cultural Norms

To examine the contribution of cultural norms to skin disclosure in a virtual setting, we examined a separate population of avatars belonging to the “Star Wars Role-Play” (SWRP) community of Second Life, one of the most vivid role-play communities of Second Life [25], [26]. Members of this community reenact the fictional universe of “Star Wars” via their avatars in the virtual space of Second Life and would thus be expected to adhere to the dress code depicted in the Star Wars movies. An additional separate sample of 108 avatar images (53 males and 55 females) was collected between July 2011 and January 2012 in randomly selected locations appearing under the “SWRP” keyword in the Second Life search engine. The avatar sampling approach was the same as described above. Only humanoid avatars that were not covered by fur were included in the analysis. The percentage of exposed skin for each of these avatars was measured according to the same methods as described for avatars originating from the general population of Second Life. A similar analysis was also conducted on 205 humanoid characters (116 males and 89 females) whose whole body clothing was clearly shown in the most recent Star Wars movie trilogy (“Episode I: The Phantom Menace”, “Episode II: Attack of the Clones”, “Episode III: Revenge of the Sith”). As many characters in the movies wear official uniforms (e.g., Clones, Storm Troopers, Queen Amidala’s servants), only one occurrence of a specific uniform was included in the analyses. Still images of these movie characters were obtained and exposed skin was measured in the same way as described above for the avatars. The percentage of exposed skin was measured in relation to the surface of the entire body as well as in relation to the surface of the body with the head and hands excluded.

### Statistical Analysis

The proportion of male and female avatars covering 75–100%, 50–74%, 25–49%, and 0–24% of their naked skin was compared using a Chi-square test. Furthermore, the difference in the percentage of exposed skin between male and female avatars was assessed using a Mann-Whitney U test. A more conservative non-parametric statistical approach was selected because analyses of the percentage of exposed skin involved bound measures (percentages). Non-parametric Spearman’s correlation analyses were used to determine if there was a relationship between body shape ratios and the percentage of naked skin disclosed by male and female avatars. Finally, a non-parametric Kruskal-Wallis test was used to statistically compare the percentage of exposed skin between males and females from the two Star Wars media. Post-hoc analyses were performed using Mann-Whitney U tests followed by a Bonferroni correction to account for multiple comparisons. All statistical analyses were conducted with the rejection level set at *p*<0.05, unless otherwise indicated.

## Results

### Avatar Skin Disclosure

**Figure 1:**
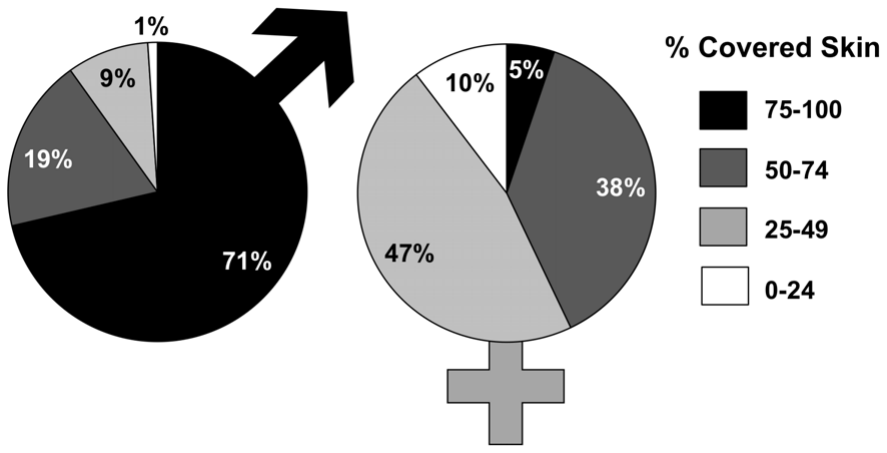
Propensity to cover skin among virtual males and females. Shown is the percentage of Second Life male and female avatars in relation to the percentage of covered skin.

The proportion of avatars that covered 75–100%, 50–74%, 25–49%, and 0–24% of their naked skin varied with the sex of the avatars (Figure 1). There was an overall significant difference in the proportion of male and female avatars in each category (χ_2_(3, N = 404) = 170.60, *p*<0.001). 71% of male avatars covered between 75–100% of their skin while only 5% of females in the same category did. Conversely, only 19% of male avatars compared to 38% of female avatars covered between 50–74% of skin, 9% of males compared to 47% of females covered between 25–49% of skin, and 1% of males compared to 10% of females covered only 0–24% of their skin.

**Figure 2:**
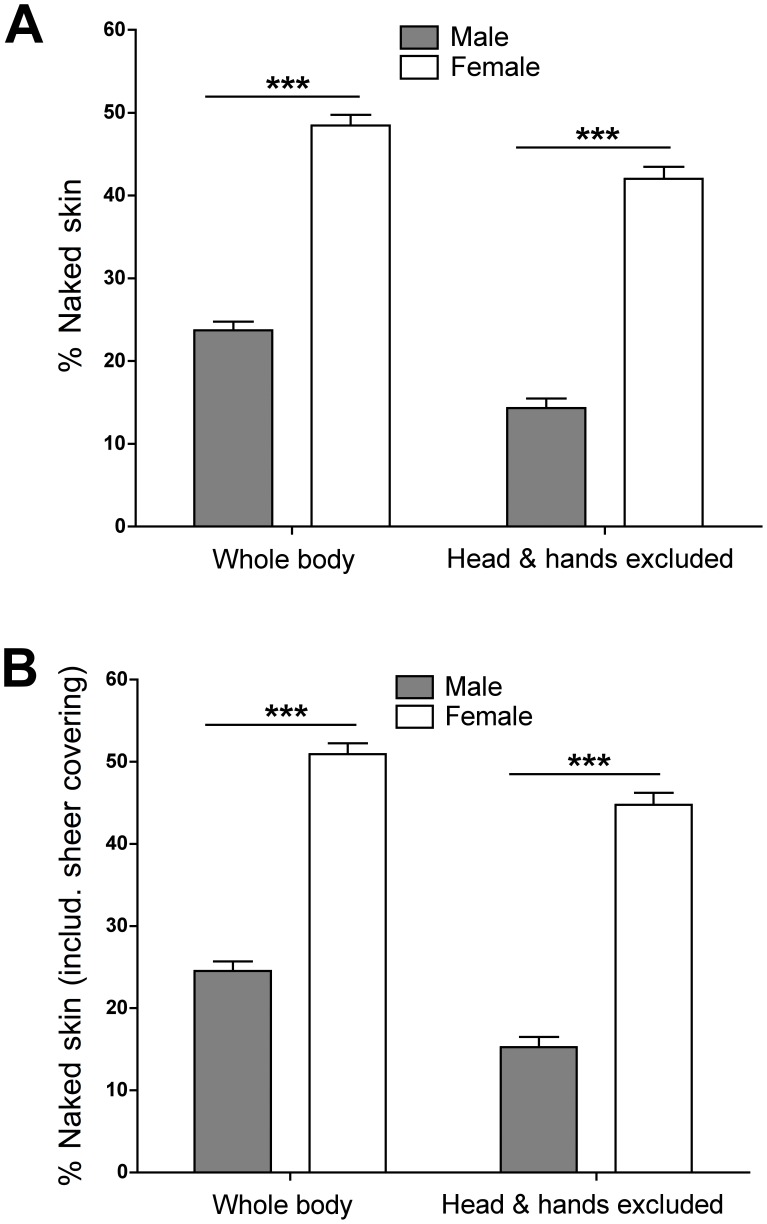
Degree of skin disclosure among virtual males and females. Shown is the percentage of exposed naked skin among male and female avatars in relation to the area of the entire body and of the body excluding the head and hands. Female avatars revealed twice as much naked skin as male avatars (A) and this difference was even more pronounced when sheer skin covering was considered (B). Results are presented as means ± SEM. ***p<0.001.

There was a substantial difference between male and female avatars in the proportion of skin exposed (Figure 2A), with female avatars exposing over twice as much skin as males (*U* = 5622.5, *p*<0.001, *r* = −0.63). This difference was just as pronounced when the head and hands, areas of skin typically exposed for communication and sensory functions, were excluded (*U* = 5621.5, *p*<0.001, *r* = −0.63). When areas of skin with sheer covering were included (Figure 2B), the differences in exposed naked skin between male and female avatars was just as large both when the whole body was considered (*U* = 5415.0, *p*<0.001, *r* = −0.63) and when the head and hands were excluded (*U* = 5419.5, *p*<0.001, *r* = −0.63).

### Contribution of Users’ Sex

**Figure 3:**
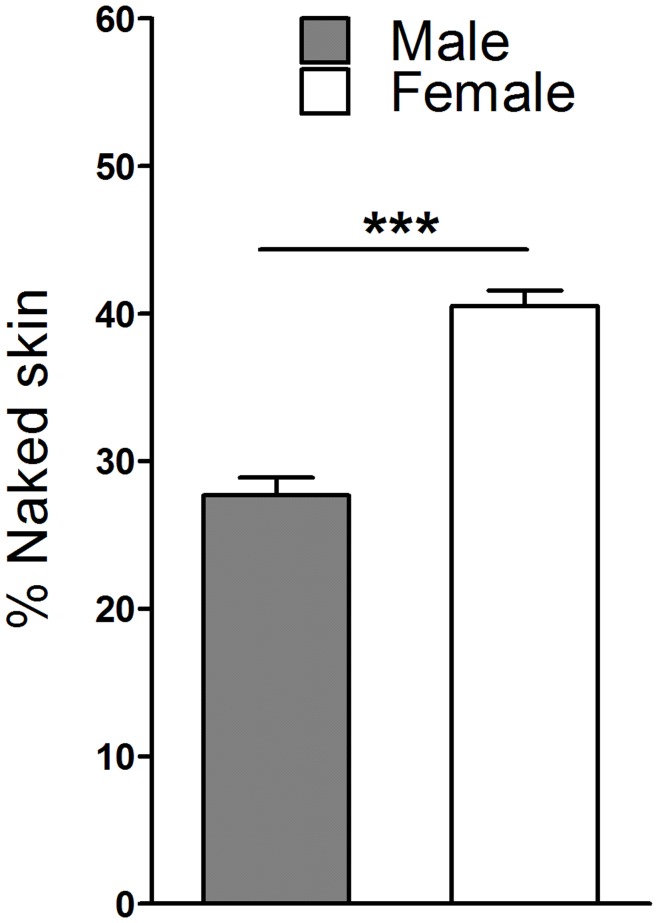
Contribution of users’ sex to skin disclosure among male and female avatars. Potential sex swapping among users was accounted for by excluding the top 25% of female avatars that exposed most skin and the bottom 25% of male avatars that exposed least skin. Even after excluding these avatars, the remaining female avatars revealed significantly more skin than remaining male avatars. Results are presented as means ± SEM. ***p<0.001.

The difference in skin exposure between male and female avatars was significant even when sex swapping was accounted for by removing from the analysis the top 25% of female avatars that exposed most skin and the bottom 25% of male avatars that exposed least skin (*U* = 5326.5, *p*<0.001, *r* = −0.46; Figure 3).

### Contribution of Body Shape

**Figure 4:**
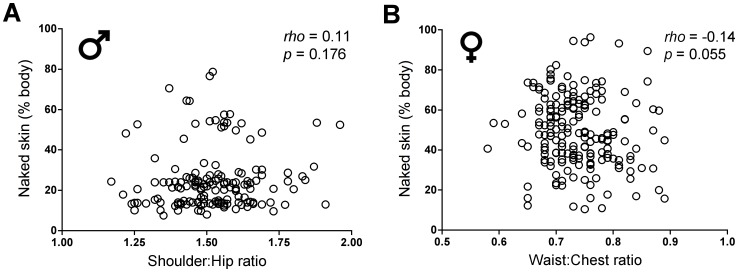
Contribution of sexualized portrayal of body shape to skin disclosure. Shown is the percentage of naked skin disclosed by male (A) and female (B) avatars as a function of body proportions related to sexual attractiveness (frontal shoulder:hip ratio in males, frontal waist:chest ratio in females). Spearman correlation analyses revealed no significant relationship between body proportions and the percentage of exposed naked skin, indicating that virtual skin disclosure is independent of any hypersexualization of avatar body shapes.

Spearman correlation analyses did not reveal any significant relationship between avatar body proportions implicated in female sexual attractiveness, specifically waist to hip and waist to chest ratios, and exposure of naked skin in either sex. Similarly, Spearman correlation did not reveal any significant relationship between avatar body proportions implicated in male sexual attractiveness, specifically waist to shoulder and shoulder to hip ratios and exposure of naked skin in either sex (results presented in Table 1). The distribution of data points for two representative body proportions, shoulder to hip in males and waist to chest in females, is shown in Figure 4.

**Table 1:** Results of Spearman correlation analyses of the percentage of exposed naked skin and sexually dimorphic body proportions.

Ratio	Male			Female		
	*rho*	*p*	*n*	*rho*	*p*	*n*
**Front**						
Waist:Hip	−0.05	0.540	151	−0.14	0.068	168
Waist:Chest	0.01	0.895	158	−0.14	0.055	184
Waist:Shoulder	−0.12	0.140	157	−0.04	0.637	169
Shoulder:Hip	0.11	0.176	151	−0.11	0.176	150
**Back**						
Waist:Hip	−0.04	0.625	137	−0.07	0.429	133
Waist:Chest	−0.12	0.178	136	−0.06	0.534	115
Waist:Shoulder	−0.11	0.206	138	−0.02	0.797	122
Shoulder:Hip	0.14	0.101	149	−0.07	0.416	124

### Contribution of Explicit Cultural Norms

**Figure 5:**
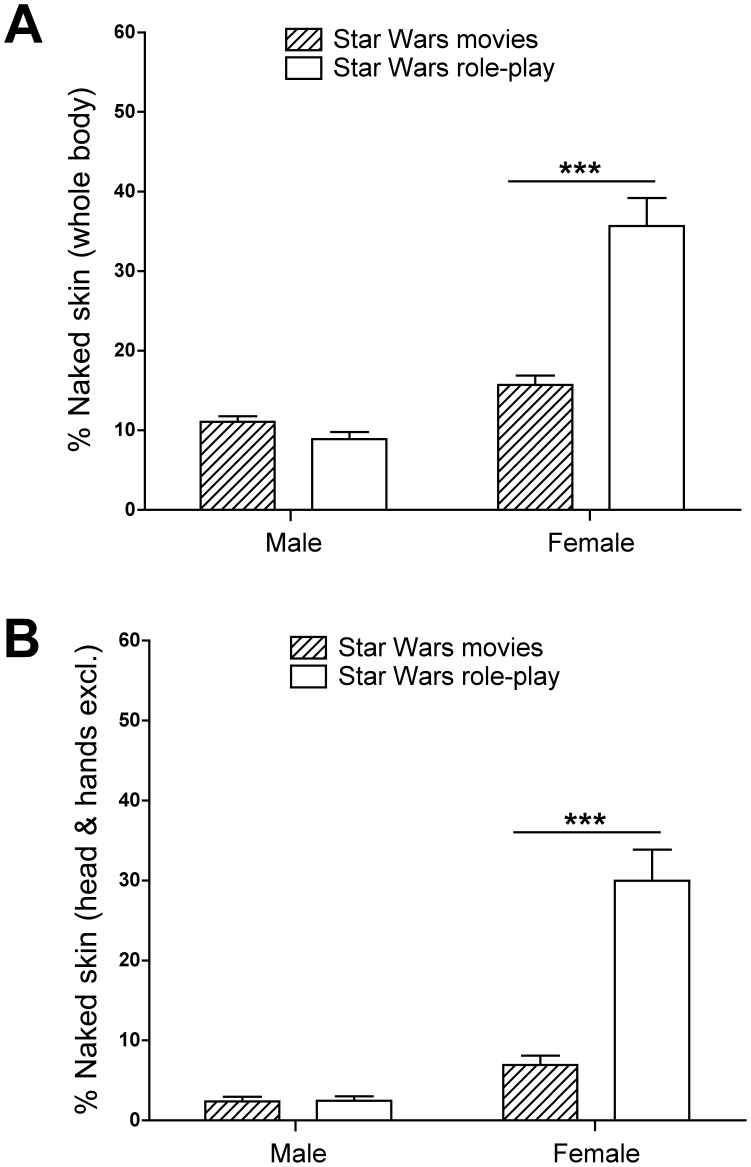
Influence of explicit cultural norms on virtual skin disclosure. Shown is the percentage of exposed skin in relation to the area of the entire body (A) and of the body excluding the head and hands (B) for avatars from the “Star Wars” role-play community and characters from the “Star Wars” movies. There was no difference between male avatars and movie characters, but female avatars revealed substantially more naked skin than female movie characters, despite being in a culturally constrained virtual setting. Results are presented as means ± SEM. ***p<0.001.

When skin disclosure was compared across four groups, male and female avatars belonging to the Second Life Star Wars Role-Play community and male and female characters in the Star Wars movies (Figure 5A), there was a significant difference between the groups in the degree of naked skin disclosed (*H*(3) = 83.12, *p*<0.001). Posthoc analyses were performed using Mann-Whitney U tests with a Bonferroni correction setting the *p* value at 0.025 to control for multiple comparisons. There was no significant difference in the percentage of exposed skin between male Star Wars Role-Play avatars and male Star Wars movie characters (*U* = 2453.5, *p* = 0.035, *r* = −0.16). However, female avatars disclosed significantly more skin than expected when compared with female movie characters, despite being in a culturally-constrained environment (*U* = 1152.5, *p*<0.001, *r* = −0.44).

A similar pattern of results emerged when the surface of the body excluding the head and hands was considered (Figure 5B). Again, there was a significant difference between the four groups in the degree of naked skin disclosed (*H*(3) = 96.10, *p*<0.001). Posthoc analyses once again revealed that there was no difference in the percentage of exposed skin between male Star Wars Role-Play avatars and male Star Wars movie characters (*U* = 2537.5, *p* = 0.063, *r* = −0.14). In contrast, female avatars disclosed significantly more skin than expected when compared with female movie characters (*U* = 950.5, *p*<0.001, *r* = −0.51).

## Discussion

In the present study we used an online multi-user 3D virtual setting, where users can interact by means of avatars, to examine the spontaneous human tendency to disclose naked skin. We observed a substantial sex difference, with female avatars disclosing twice as much naked skin as male avatars, even in the context of explicit cultural norms promoting less revealing attire. This sex difference was evident not only when the surface area of the entire body was considered, but also when parts of the body that are typically exposed for sensory and communication purposes (i.e., head and hands) were excluded. The findings thus capture for the first time evidence of a sex difference in human skin disclosure outside of external climatic, environmental, and physical constraints.

One limitation of this study is that the sex of the users behind the avatars is unknown. We can only speculate whether the observed sex difference in skin disclosure among avatars reflects the sex-dependent skin disclosure tendencies of the users. However, previous work demonstrates that the representation of males and females among the population of Second Life users is fairly equal, and that males are not over-represented in this virtual world, as is the case in other video game-based virtual worlds [19]. As well, less than 25% of users of online virtual worlds, including Second Life, have been found to take on the opposite sex for their avatars [19]. Therefore, a large proportion of avatars do reflect the sex of their user. Given the magnitude of the observed effect in the skin disclosure of female avatars, we expect that female users behind female avatars contribute to a large portion of this effect. Indeed, this hypothesis is supported by our statistical analysis demonstrating a robust sex difference even after the putative influence of sex swapping was accounted for by excluding the top 25% of female avatars that exposed the most naked skin and the bottom 25% of male avatars that exposed the least naked skin.

One explanation for this sex difference in skin disclosure could be hypersexualization of female virtual representations. Indeed, this is commonly observed in video game characters, where females are more likely to be portrayed in revealing attire and with unrealistic body proportions [20], [27]. An important difference between characters in video games and avatars in user-generated virtual settings such as Second Life is that video game designers typically generate the appearance and clothing of video game characters, whereas the users themselves generate the appearance and clothing of their avatars. If skin disclosure was indeed related to hypersexualization of avatars by Second Life users, body proportions associated with greater sexual attractiveness would be related to the degree of disclosed skin. However, this was not the case as no association was found between body proportions and skin disclosure in either sex. Therefore, our analysis of the body proportions of avatars strongly suggests that the spontaneous user-generated tendency for female avatars to reveal more skin than male avatars is independent of hypersexualization.

In comparison to classical multi-user video game settings, members of the virtual world of Second Life on the whole do not adhere to any explicit cultural clothing norms for avatars. However, there are many sub-communities in Second Life that engage in role-play and their members adhere to clothing norms related to the theme of the role-play [25], [26], [28]. In order to examine the influence of cultural norms on skin disclosure within the virtual setting, we assessed the pattern of skin disclosure among avatars belonging to the popular Star Wars Role-Play community [25], [26]. Avatars reenacting the fictional culture of the Star Wars universe are expected to dress similarly to characters portrayed in the movies, hence also displaying comparable amounts of naked skin in their attire. Indeed, male avatars did not differ in the degree of exposed naked skin from male characters portrayed in the most recent Star Wars trilogy. However, female avatars surprisingly disclosed substantially more naked skin than would have been expected based on the analysis of female movie characters. In other words, the attire chosen for male avatars corresponded on average with the cultural norms of male attire established in the movies. Conversely, the attire chosen to dress female avatars was on average more revealing compared to the cultural norms of female attire established in the movies. These findings indicate that the observed propensity of virtual females to reveal naked skin persists despite explicit cultural norms promoting less revealing attire.

Nevertheless, implicit cultural influences on skin disclosure within the virtual setting of Second Life cannot be discounted. The real-life culture of users may influence the way they dress their avatars. As the prevalence of Internet access and its use is still highest in North America, Europe, and Australia [29], the majority of users of Second Life likely originate from these parts of the world, where Western culture prevails. The observed findings may thus reflect this cultural influence to a certain degree. Similarly, revealing skin may be a reflection of prevalent Second Life cultural trends promoted by particular clothing designers. However, users of Second Life have easy access to a vast selection of user-generated clothing reflecting both the general and role-play communities, including clothing related to particular role-play settings where some women are expected to cover their naked skin and males are expected to display more skin (e.g., the Gorean community, [28]). As well, both freely available and purchased clothing can typically be modified to be more or less revealing (e.g., length of sleeves, depth of cleavage). Furthermore, the types of clothing available in Second Life are subject to user-driven economic variables, such as customer demand for certain products [15]. Thus, available clothes may indeed reflect the wishes of the community. Although further work is required to tease apart the contribution of these variables, the wide variety of clothing available on the Second Life market combined with our large random sample of avatars suggest that the sizable difference in skin disclosure between male and female avatars more so reflects an emergent behavioral tendency rather than a particular cultural influence.

An important question that arises from these findings is what influences the behavioral tendency for female avatars to disclose more naked skin, beyond potential cultural influences. The most parsimonious explanation relates to naked skin as a display of sexual attractiveness. Previous work demonstrates that males rate females as more attractive when they display more naked skin [30], with areas of naked skin on the female body attracting preferential visual attention from males compared to clothed areas (reviewed in [31]). Furthermore, females are aware of the function of their clothing as a sexual signal [31], while males use clothing as a sign of a female’s intended long or short-term mating strategy, with a short-term strategy associated with more revealing attire [32], [33]. Thus, the tendency of female avatars to display naked skin in the virtual world could reflect the desire to accentuate sexuality and attract attention. However, this explanation is not completely satisfactory, given that there was no association between body proportions related to sexual attractiveness and the degree of skin disclosure. Another factor that may contribute to greater skin disclosure in females relates to the role of exposed skin in tactile contact [8], [9]. Previous studies suggest that, at least in some contexts, females use touch more than males in social interactions and in communicating prosocial emotions [34]–[36]. Skin disclosure by female avatars could be indicative of cross-modal compensation for the lack of tactile input in the virtual context. Appearance of exposed naked skin could enhance the perceived representation of interpersonal tactile contact when such contact is visualized in the virtual setting. Indeed, inter-avatar contact is common in Second Life and many regions include free animations of interpersonal interactions, such as couple poses and activities that include dancing, embracing, kissing, and various other types of intimate exchanges. In line with this hypothesis, compensatory behavior among female avatars in the virtual environment was previously observed in the context of the massively multiplayer role-playing game World of Warcraft [37].

Overall, human visual body presentation plays an important role in the way that individuals are perceived by others [7], [38]. Indeed, visual perception of the human body is facilitated by specialized neural mechanisms that are sensitive to the degree of nudity [3]. Thus, the observed spontaneous behavioral tendency towards greater skin disclosure among virtual females has implications for further understanding how sex-specific visual body presentation guides human social interactions in both virtual and real spaces [39]. As our observational approach enables us only to make inferences about the behavioral tendencies of the human users behind the avatars, further experimental studies are still needed to examine the role of sex in human skin disclosure more directly. Nevertheless, multiuser 3D virtual settings can provide a useful new realm to explore such questions regarding human body presentation in conditions that are unconstrained by the climatic, environmental, and physical limitations of the real world.
